# A 39 years old woman with thoracic endometriosis presents with recurrent catamenial Pneumothorax: A case report

**DOI:** 10.1016/j.ijscr.2025.111903

**Published:** 2025-09-05

**Authors:** Armin Amirian, Seyed Mehdi Ghazanfari, Parviz Mardani, Samane Gorjizade, Mana Moghadami

**Affiliations:** a Thorasic and Vascular Surgery Research Center, Shiraz University of Medical Sciences, Shiraz, Iran; b General Surgery Resident, School of Medicine, Shiraz University of Medical Sciences, Shiraz, Iran; c Clinical Education Research Center, Shiraz University of Medical Sciences, Shiraz, Iran

**Keywords:** Catamenial pneumothorax, Thoracic endometriosis, Menstruation, Spontaneous pneumothorax, Case report

## Abstract

**Introduction and importance:**

Recurrent catamenial pneumothorax (RCP) is an infrequent medical condition predominantly affecting women of reproductive age. It is characterized by the repeated occurrence of pneumothorax, resulting in either partial or complete lung collapse due to air or gas in the pleural cavity. The distinctive feature of this condition is its association with the menstrual cycle, with symptoms typically manifesting within 72 h following the onset of menstruation. While catamenial pneumothorax is recognized as the most prevalent form of thoracic endometriosis syndrome, recurrent catamenial pneumothorax remains uncommon and lacks comprehensive characterization in the medical literature. Consequently, the understanding of this condition's underlying mechanisms and contributing factors is limited.

**Case presentation:**

The present report describes a thirty-nine-year-old woman with recurrent pneumothorax. Video-assisted thoracoscopic surgery (VATS) with pleurodesis reveals diffuse parietal pleura and diaphragm inflammation. Notably, red nodules and pores were observed in the central region of the right hemidiaphragm, providing compelling evidence supporting thoracic endometriosis and catamenial pneumothorax as the underlying cause. A pneumonolysis, wedge resection, partial pleurectomy, and pleural abrasion were performed, followed by applying a mesh graft to the diaphragm. After surgery, the patient was referred for hormonal therapy and remained symptom-free during follow-up visits.

**Clinical discussion:**

This case highlights the importance of recognizing catamenial pneumothorax. For treatment, both surgical intervention and hormonal therapy are essential.

**Conclusion:**

RCP should be considered as one of the differential diagnoses in reproductive-aged women presenting with repeated spontaneous pneumothorax, particularly during menstrual periods.

## Highlights

• Recurrent catamenial pneumothorax is a rare manifestation of the Thoracic Endometriosis syndrome.
• Surgical intervention with hormonal therapy is recommended in the treatment of Recurrent catamenial pneumothorax.
• This case underscores the importance of considering thoracic endometriosis in women with cyclical pneumothorax and supports a multidisciplinary management approach.

## 1 Introduction

Endometriosis is a chronic disease affecting multiple organs in women, typically within the pelvic cavity, with significant health burdens due to its recurrent nature, requiring frequent medical visits and treatment [1]. It is estimated to affect 6 to 10 % of women of reproductive age [2]. Endometriosis arises from the abnormal growth of endometrial tissue in various body parts, primarily observed in the pelvic cavity, but can also be found in other parts of the body [1,3,4]. Among people experiencing endometriosis, about 12 % may experience endometriosis in non-gynecological organs [2].

Although pelvic endometriosis is the most common localization, endometrial tissue can go further in the thoracic cavity, recognizing the Thoracic Endometriosis syndrome (TES) [5]. Thoracic endometriosis, although rare, is often underreported [6]. The clinical presentation of TES varies from catamenial pneumothorax (CP), catamenial hemothorax, catamenial hemoptysis to pulmonary nodules, occurring during the menstruation period [5].

CP refers to the occurrence of spontaneous pneumothorax during menstrual periods, typically within the first 72 h after the onset of menstruation [3,7,8]. It is reported to be the most prevalent manifestation of thoracic endometriosis syndrome, representing 7.3—36.7 % of spontaneous pneumothorax in women of reproductive age [9]. The systematic review studying the pneumothorax estimated CP as 7.3—36.7 % of spontaneous pneumothorax in women of reproductive age [9]. Herein, we report a rare case of recurrent catamenial pneumothorax. The purpose of describing this case is to raise awareness among clinicians about the potential difficulty in diagnosing thoracic endometriosis, given its diverse clinical presentations and imaging characteristics. This case report has been reported in line with the SCARE 2025 guidelines [10].

## 2 Case presentation

A thirty-nine-year-old woman from Iran with a past medical history significant for hypothyroidism presented to the hospital complaining of shortness of breath. The dyspnea had started two days before admission, following a previous hospitalization approximately seven days earlier for the same symptom. At that previous hospitalization, pneumothorax was diagnosed, and a chest tube was inserted and subsequently removed after three days with resolution of the symptoms and pneumothorax. However, the patient experienced a recurrence of dyspnea within two days of discharge. No known allergies, alcohol or tobacco use, or history of prescription or illegal drug use were identified, eliminating these factors as potential contributors to the patient's symptoms. The patient did not have any recent history of chest trauma and presented as hemodynamically stable without signs of acute distress. She did not report a prior diagnosis of pelvic endometriosis, and pelvic evaluation was performed to rule out pelvic involvement; the evaluation was normal.

On examination, vital signs were within normal limits, and no tracheal deviation was observed.

**Fig. 1:**
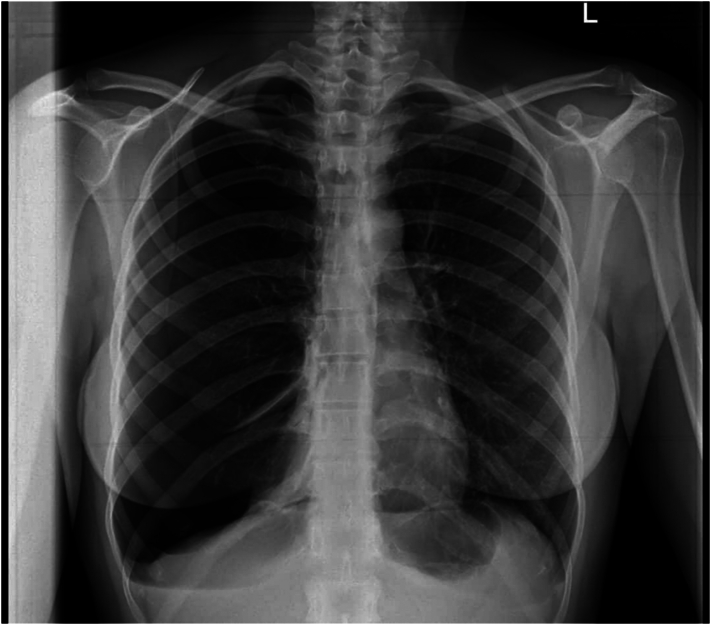
Chest X-Ray showed pneumothorax on the right side.

**Fig. 2:**
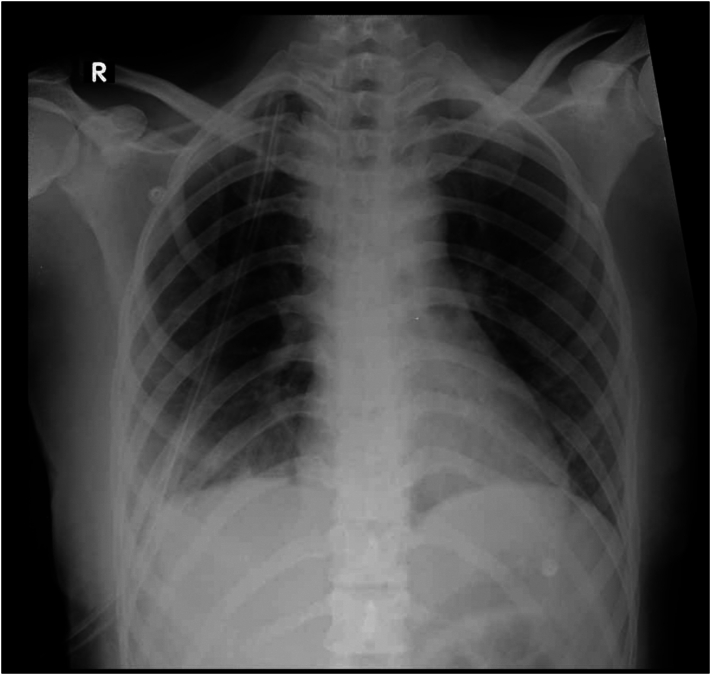
Chest tube was inserted.

On chest auscultation, clear breath sounds were noted on the left side, while decreased breath sounds were detected on the right side. A chest X-ray was performed due to decreased breath sounds, revealing the presence of a right pneumothorax (Fig. 1). Subsequently, a chest tube was inserted on the right side to alleviate the condition (Fig. 2). To further investigate any underlying parenchymal lung disease causing the pneumothorax recurrence, a spiral computed tomography (CT) scan of the chest was indicated, after chest tube placement, confirming the chest tube was well positioned in the pleural cavity and showing no evidence of bullous emphysema. It showed a right pneumothorax with lung collapse.

**Fig. 3:**
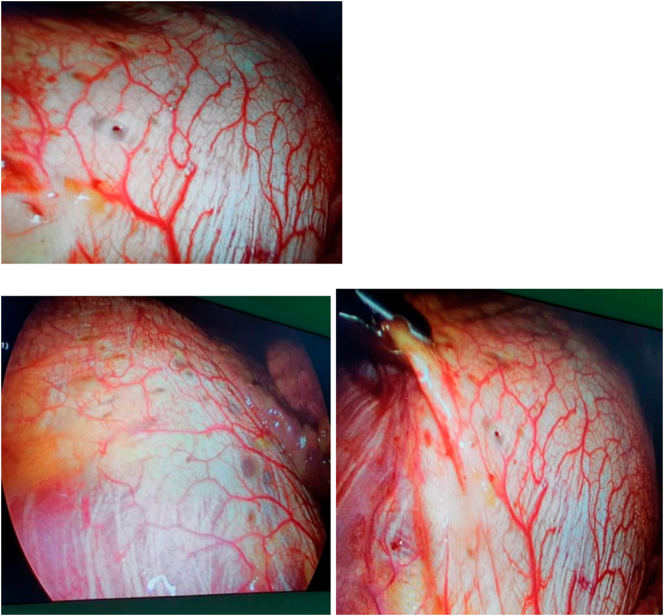
Intraoperative images of thoracic endometriosis.

**Fig. 4:**
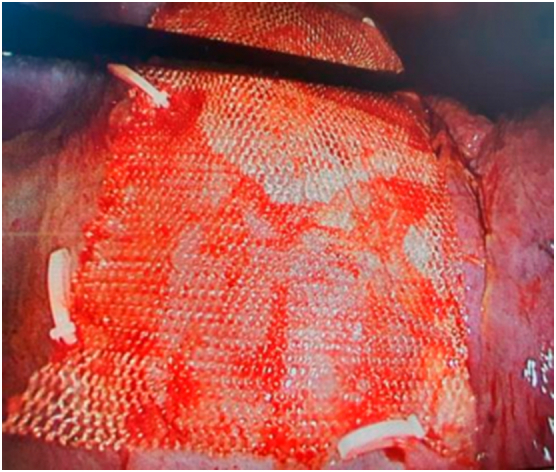
Mesh graft and hemlock were inserted.

To address the pneumothorax, the patient underwent VATS. During the procedure, diffuse parietal pleura and diaphragm inflammation were observed. Notably, red nodules and pores were detected in the central part of the right hemidiaphragm, providing strong evidence in favor of thoracic endometriosis (Fig. 3). Pneumonolysis was performed, and a wedge resection of the apical segment of the right upper lobe was conducted using two linear endo staplers (Number 60). Subsequently, a partial parietal pleurectomy of the upper hemithorax and pleural abrasion of the lower hemithorax were performed. Thorough irrigation and hemostasis were performed, leading to a complete lung expansion without any evidence of air leakage. As a part of the surgical management, a mesh graft was utilized to cover the central portion of the diaphragm, which was fixed with a hemlock (Fig. 4). Following the surgical intervention, the patient was referred for post-surgical hormonal therapy, gonadotropin-releasing hormone (GnRH a) to suppress endometrial activity.

**Fig. 5:**
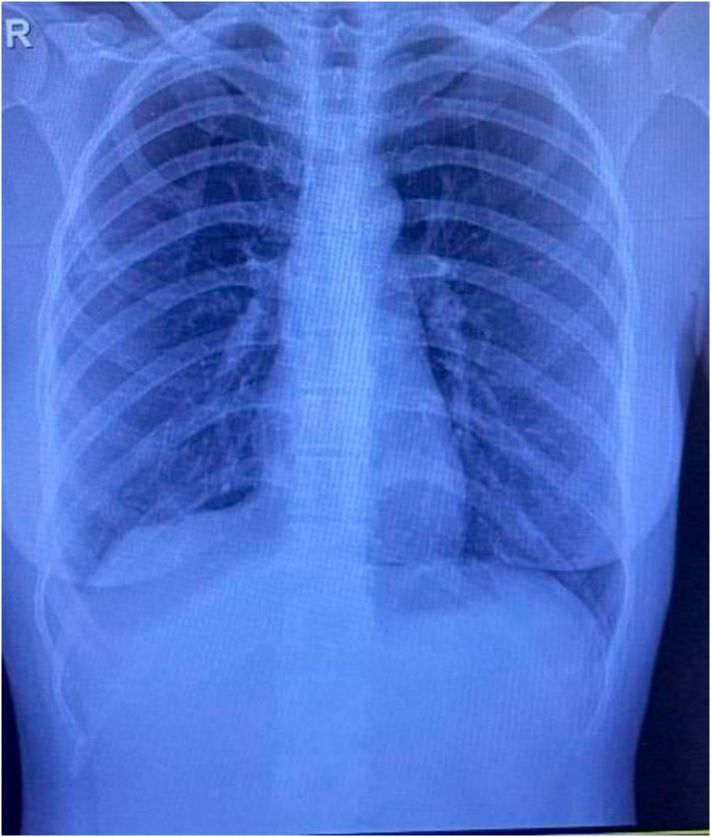
Chest X Ray one month after the surgery.

**Fig. 6:**
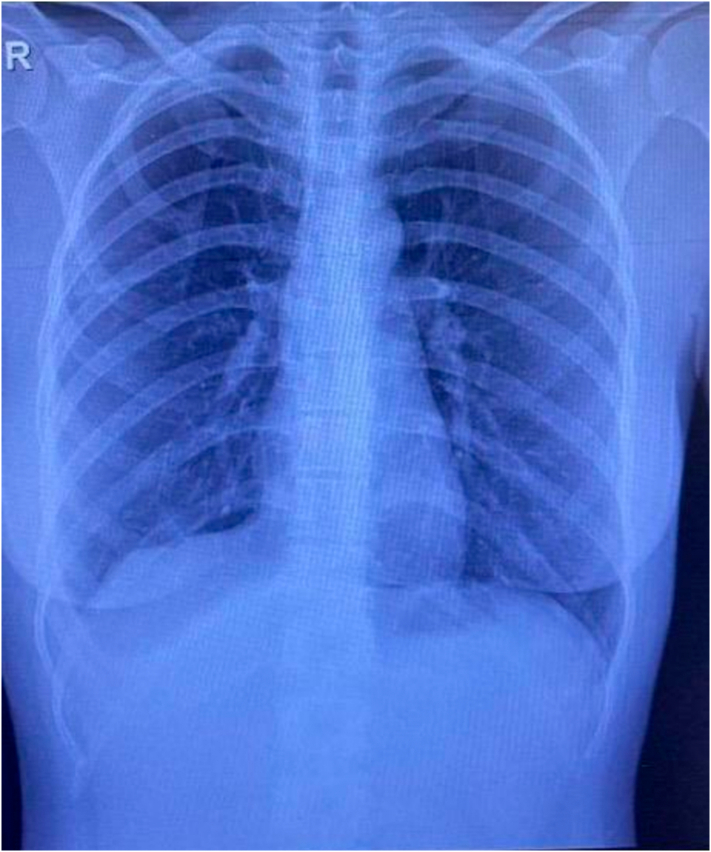
Chest X-Ray three months after the surgery.

A biopsy taken from a solitary nodule confirmed the diagnosis of thoracic endometriosis, including endometrial-like glands and stroma, along with hemosiderin-laden macrophages. These findings confirmed the diagnosis of thoracic endometriosis. During the patient's follow-up visit, approximately one month after the surgical intervention, no symptoms of dyspnea or other related issues were reported. Similarly, during the subsequent visit three months later, the patient remained free of dyspnea or any notable difficulties for around three months post-operation. Chest X-rays were conducted, revealing the absence of pneumothorax. (Fig. 5, Fig. 6).

## 3 Discussion

Recurrent spontaneous pneumothorax associated with the menstrual period was first described by Maurer et al. in 1958 [11]. Later in 1972, Lillington et al. named this condition catamenial pneumothorax [12]. Catamenial pneumothorax as the clinical presentation of TES is a rare condition with a high rate of recurrence [13]. While thoracic endometriosis was previously considered a rare manifestation of pelvic endometriosis, clinicians must be aware of this condition and catamenial pneumothorax [14,15].

The pathophysiology of TES has not been fully understood. However, the most accepted theory that accounts for all of the TES's syndrome is the retrograde menstruation theory, well known as Sampson's theory. It proposed retrograde migration of the endometrial tissue throughout the fallopian tubes into the peritoneal cavity, then along the right paracolic gutter to reach the diaphragm as the main mechanism in this disease. Through small defects in the diaphragm, this tissue may enter the thoracic cavity [2]. Other proposed pathophysiological theories are coelomic metaplasia, lymphatic and hematogenous spread, prostaglandin-theory and diaphragmatic theory of air passage [2,16].

Symptoms of TES vary from pleuritic chest pain and cough to shortness of breath and hemoptysis. The right hemithorax is involved in most cases [2]. Endometrial tissue within the peritoneum often adheres to this directional flow, frequently affecting the right hemidiaphragm. The falciform ligament's presence restricts tissue migration to the left side. Additionally, variations in intraperitoneal pressure during respiration cause the right hemidiaphragm to contract, creating a “piston effect” in contrast to the liver [17]. Furthermore, congenital defects in the diaphragm are more commonly observed on the right side.

Haga et al. emphasize four clinical factors that distinguish catamenial pneumothorax from spontaneous pneumothorax: right-sided pneumothorax, a previous diagnosis of pelvic endometriosis, an age range of 30–40 years, and a lack of smoking history. These clinical presentations demonstrate a strong predictive value in identifying catamenial pneumothorax instead of spontaneous pneumothorax. While earlier studies indicated that 3–6 % of spontaneous pneumothoraxes in women met the criteria for catamenial pneumothorax, recent articles propose a considerably higher rate, reaching up to 35 %, among patients who undergo surgical intervention [18].

In this study, the case of recurrent catamenial pneumothorax in young women with a history of two times spontaneous is a rare medical condition that is difficult to diagnose, characterized by the repetitive occurrence of spontaneous pneumothorax in women, which is linked to their menstrual cycle [14]. This condition's primary cause is the presence of endometrial tissue in the thoracic cavity, known as thoracic endometriosis. The situation is remarkable in its rarity, as such occurrences are seldom encountered or documented.

Diagnostic evaluation is started with a Chest X-ray to confirm the presence of the pneumothorax; CT scans may show collapsed lung segments. In our case, both the Chest X-ray and CT scan showed pneumothorax. Magnetic resonance imaging (MRI) can help detect diaphragmatic defects and air-filled bubbles. In addition, abdominal MRI can be used in the diagnosis of endometriosis and TES [4]. Bronchoscopy has limited diagnostic use in endometriosis as the lesions lie more peripherally rather than airway-based; however, it can help rule out other causes, such as unexplained hemoptysis or other pathologies [19].

VATS, due to its minimally invasive nature, is widely accepted as the gold standard diagnostic and therapeutic approach [2]. However, in some cases, an inaccessible diaphragmatic muscle-sparing thoracotomy may be needed to repair diaphragm defects [2,20]. To ensure optimal visibility of characteristic signs and pathological findings, scheduling surgery during the menstrual period is advisable [21]. In most cases, thoracoscopic surgery involves resection of any visible bullae or abnormal lung tissue, parietal pleurectomy if parietal pleura is involved, and partial resection of the diaphragm in the presence of deep diaphragmatic lesions [5,22].

In the management of diaphragmatic lesions of TES, the choice between resection, plication, or reconstruction depends on the distribution and the extent of the lesions. As reported in the literature, the resection of diaphragmatic tissue is preferred over plication. In cases with more widespread lesions, plication may be the choice. In addition, mesh reinforcement has been shown to have a good outcome depending on the intraoperative outcomes [4].

In our case, VATS was performed with the described procedures, resulting in successful treatment of the condition. The lung re-expanded and filled the thoracic cavity without any air leakage. The patient underwent pneumonolysis, wedge resection of the affected lung tissue, partial pleurectomy, and pleural abrasion. A mesh graft was applied to reinforce the diaphragm. Furthermore, the diagnosis of endometriosis was confirmed through histopathological examination. In this case, a history of endometriosis and the pathological findings further support this diagnosis.

Hormonal therapy after the surgery, mostly GnRH agonist, is recommended to suppress estrogen production and reduce the recurrence [22]. In our case, the GnRH agonist was also initiated after the surgery. Literature reported the recurrence of CP about 8 to 40 %; however, in our case, the patient had no recurrence [23].

Overall, recurrent catamenial pneumothorax is a rare condition; only a few cases have been reported in the literature. Catamenial pneumothorax should be considered in a young patient experiencing dyspnea and chest pain. To diagnose and treat pulmonary endometriosis promptly, the involvement of a multidisciplinary team comprising pulmonologists, thoracic surgeons, gynecologists, pathologists, and radiologists is crucial.

## 4 Conclusion

In summary, recurrent catamenial pneumothorax is a rare condition that warrants consideration in young women who experience dyspnea and chest pain related to their menstrual period. It is essential to confirm the diagnosis through histopathologic examination, specifically confirming the presence of endometriosis. The literature reports limited cases of catamenial pneumothorax, underscoring the need for increased awareness among healthcare professionals. A multidisciplinary approach involving various specialists is crucial to ensure accurate diagnosis and timely treatment. Further research and case studies are necessary to enhance our knowledge of this condition and improve patient outcomes.

## 5 Strengths and limitations

### 5.1 Strengths

This case review most of the relevant literature of RCP and shows how surgery plus hormonal therapy can help. The clear findings on VATS and the teamwork between surgery and gynecology made the diagnosis and treatment more effective.

### 5.2 Limitations

It is just one case and it decreases the generalizability of the findings.

## Consent for publication

The patient in this case report has provided written informed consent was obtained from the patient for publication of this case report and any accompanying images. A copy of the written consent is available for review by the Editor-in-Chief of this journal.

## Ethics approval and consent to participate

The local Clinical Research Ethics Committee does not need approval for case reports.

## Author contributions

AA, PM, SMGH, and SG performed clinical management of the patient. SMGH and MM conducted the literature survey and wrote the manuscript. AA, PM, and SG completed the literature survey and edited the manuscript. All authors read and approved the final manuscript.

## Funding

No funding was received for this case report.

## Declaration of competing interest

The authors declare that they have no competing interests.

## Data Availability

Data is available on request due to privacy and ethical restrictions.
